# Treasured but not measured ?: Impact of the COVID-19 pandemic on physical literacy in children and adolescents

**DOI:** 10.1016/j.jesf.2025.200434

**Published:** 2025-12-16

**Authors:** John J. Reilly, Kerry Robertson, Farid Bardid

**Affiliations:** a University of Strathclyde Department of Psychological Sciences and Health, Glasgow, Scotland, United Kingdom; b University of Strathclyde Institute of Education, Glasgow, Scotland, United Kingdom

**Keywords:** Child, Adolescent, Youth, Physical literacy, Movement restrictions, Lockdown

## Abstract

**Objective:**

Physical literacy is treasured because it underpins participation in physical activity and sport in children and adolescents. Physical literacy might have declined following COVID-19 movement restrictions, but whether such a decline took place is uncertain. This study aimed to examine if a post-COVID-19 decline in physical literacy occurred in children and adolescents.

**Methods:**

A systematic review, registered on PROSPERO CRD42025646499 in February 2025, was used to assess changes in physical literacy following the COVID-19 pandemic in healthy, typically developing 3–18 year olds. Searching in June 2025 used 7 databases: Scopus, Web of Science, SPORTDiscus, PsychINFO, CINAHL, PubMed, Sports Medicine & Education Index and a grey literature search in Global Think Tanks.Risk of bias assessment used the Effective Public Health Practice Project (EPHPP) instrument.

**Results:**

Only one eligible study was identified, which reported declining physical literacy in 8–14 year olds in Canada between 2019 and 2020.Evidence quality was moderate as assessed using the EPHPP.

**Conclusion:**

The impact of COVID-19 movement restrictions on physical literacy in children and adolescents could not be assessed due to lack of evidence. The lack of evidence on such an important topic is a valuable finding in itself. Understanding trends in physical literacy will require greater monitoring, and the inclusion of physical literacy measurement in public health surveillance. If physical literacy is really treasured it should be measured.

## 1 Introduction

Physical literacy is an integrative construct comprised of multiple domains: cognitive; physical; behavioural; psychological/affective; social.^1^^,^^2^ It is considered to be foundational for physical activity in childhood and adolescence.^3^^,^^4^ Higher child and adolescent physical literacy is associated with health benefits.^5^ Despite its importance, levels of physical literacy in the general population of children and adolescents might be low internationally,^6^^,^^7^ consistent with low levels of physical activity.^8^

Movement restrictions introduced to mitigate the COVID-19 pandemic had substantial adverse impacts on levels of physical activity^9^ in children and adolescents, and motor competence also declined post-pandemic.^10^ These changes might have reduced physical literacy, but that is uncertain. Future infectious disease pandemics are considered inevitable by the World Health Organization (WHO) and many national governments, and so understanding COVID-19 impacts on physical literacy should inform pandemic control measures in future. The primary aim of the present study was therefore to test the hypothesis that physical literacy declined in children and adolescents following the pandemic. The secondary aim was to highlight gaps and weaknesses in the evidence.

## 2 Methods

### 2.1 Literature searching, Screening and study selection

Methods and reporting were based on the PRISMA guidance^11^ (Appendix A) and the protocol was registered on the February 4, 2025. PROSPERO CRD42025646499.

The literature search was carried out with a specialist librarian on June 23, 2025 in seven relevant electronic databases: Scopus, Web of Science, SPORTDiscus, PsychINFO, CINAHL, PubMed, Sports Medicine & Education Index. A grey literature search was carried out in Global Think Tanks on the same day. Searching and inclusion/exclusion criteria were based on the PICOS framework-population (apparently healthy, typically developing, individuals age 3.0–18.9 years), intervention (COVID-19 pandemic movement restrictions), comparison (pre-post COVID change in physical literacy), outcomes (any measure described by the authors as physical literacy so long as it included at least 3 physical literacy domains following the approach of Barnett et al.^12^ and Bailey et al.^1^). Studies were included if they had pre- and post-COVID data on physical literacy, in general population samples, excluded if they did not measure physical literacy as a composite of at least 3 domains, if measures were made in samples with chronic disease/disability, in those under 3 or over 18 years of age. Search terms and syntax were derived from other recent systematic reviews of physical literacy (Appendix B). The reference list from the eligible study was checked for other relevant studies. Study authors for one paper were contacted to check the eligibility of that paper.

### 2.2 Data extraction

Data were extracted from the eligible study using a data extraction table. Authors were contacted to provide additional data for the eligible study.

### 2.3 Data analysis, summary and synthesis

Only a single eligible study was identified-so findings were summarised narratively.

### 2.4 Risk of bias assessment

Two authors assessed risk of bias independently, checked by a third author. The Effective Public Health Practice Project^13^ was used, with the relevant domains being selection bias, study design, validity and reliability of study methods, participant attrition.

## 3 Results

**Fig. 1:**
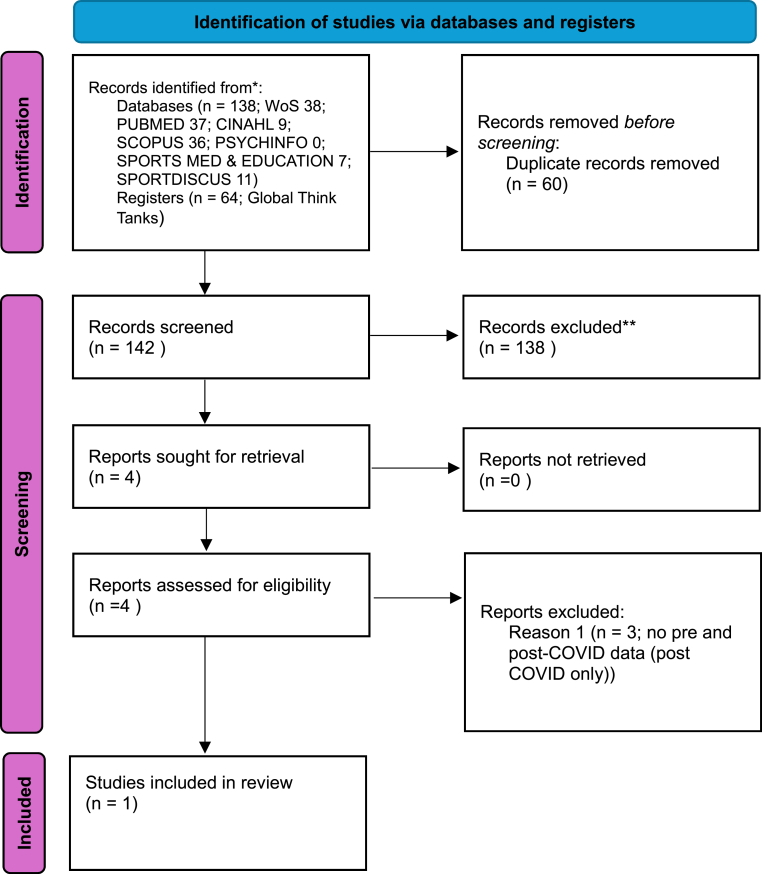
Prisma flow diagram.

Fig. 1 is the PRISMA Flow Diagram. Only 1 study was eligible for inclusion^14^ and since no quantitative synthesis was possible a brief narrative synthesis is provided here.

Houser et al.^14^ measured changes in physical literacy in 8–14 year olds from Canada (n 160), with baseline measures in May/June of 2019, repeated 12 months later in the same individuals. Physical literacy was measured using the PLAYself instrument (3 domains: environmental participation; valuing literacies; self description of Physical Literacy). In addition 182 parents completed PLAYparent assessments of their child's physical literacy, with domains of competence, confidence, understanding, environmental participation. Overall physical literacy declined significantly (p < 0.001) as measured by the PLAYparent with medium effect size (Cohen's d −0.56). There was also a statistically significant decline (p = 0.03) in overall physical literacy as measured by PLAYself, with a small effect size (Cohen's d −0.16). Using the EPHPP instrument the overall rating of the eligible study from Houser et al.^14^ was moderate, with assessments of the individual components of the EPHPP as follows: Selection Bias-moderate; Design-strong; Validity and Reliability of Methods-strong; Sample Attrition/Loss to Follow up-weak.

## 4 Discussion

The present study shows that the published evidence on changes in child and adolescent physical literacy following the COVID-19 pandemic is extremely limited. The null finding in the present study is valuable in that it shows that the widely acknowledged importance of physical literacy is not being matched by efforts to monitor levels and trends in population physical literacy.

Higher population levels of physical literacy might protect against the impact of movement restrictions on physical activity as suggested by one study,^15^ so future pandemic preparation might reasonably include efforts to improve physical literacy, especially given the other health benefits^5^ and underpinning role in sport and physical activity participation.^3^^,^^4^^,^^16^ Physical literacy measurement is widespread internationally, in research and in education, but the present study suggests that there may be widespread *measurement* but limited *surveillance* of physical literacy. Publishing of currently unpublished data might permit testing the hypothesis that physical literacy declined following the COVID-19 movement restrictions, and near-absence of published evidence on post-COVID-19 changes in physical literacy is not evidence of absence.

The near-absence of evidence in the present study reflects wider global problems with surveillance of many important child and adolescent health behaviours and health outcomes.^8^^,^^17^ Surveillance is not just a passive data gathering exercise but tends to make otherwise invisible public health problems visible, leading to policy and practice changes.^18^ Measurement methodology should not be a major barrier to surveillance.^12^ Carl et al.^19^ have also suggested that barriers to more widespread surveillance of physical literacy exist but should not be insurmountable. Thailand provides a rare international model of surveillance, with post-COVID-19 pandemic embedding of physical literacy measurement in national surveys.^7^

The present study has a number of strengths. It is the only systematic review of changes in physical literacy pre-post COVID-19 in children and adolescents and used recommended methods in both review conduct and reporting. Possible effects of movement restrictions on physical literacy in those with disability and/or chronic disease are important but could not be examined in the present study and merit further investigation. The present study was also limited to children and adolescents. COVID-19 pandemic movement restriction impacts on adult physical literacy were beyond the scope of the present study. More extensive searching for grey literature might also have identified additional eligible evidence.

In conclusion, widespread international acknowledgement of the high importance of physical literacy has not translated into monitoring of secular trends by researchers, or into public health surveillance. If physical literacy is really treasured it should be both measured^20^ and monitored.

## Data availability statement

Data used in the present study are available from the corresponding author upon reasonable request.

## Author contribution statement

Reilly: Conceptualisation; Data Curation; Formal Analysis; Methodology; Investigation; Project Administration; Supervision; Validation; Writing Draft.

Robertson: Conceptualisation; Data Curation; Formal Analysis; Investigation; Methodology; Writing-Review and Editing.

Bardid: Conceptualisation; Data Curation; Formal Analysis; Methodology; Supervision; Validation; Writing- Review and Editing.

## Funding/support statement

This study had no specific funding but the authors were funded by the Scottish Funding Council.

## Declaration of competing interests

The authors have no competing interests to declare.

## Figures and Tables

**Table:** 

Section and Topic	Item #	Checklist item	Location where item is reported
TITLE			
Title	1	Identify the report as a systematic review.	In Abstract
**ABSTRACT**			
Abstract	2	See the PRISMA 2020 for Abstracts checklist.	See Abstract
**INTRODUCTION**			
Rationale	3	Describe the rationale for the review in the context of existing knowledge.	P2
Objectives	4	Provide an explicit statement of the objective(s) or question(s) the review addresses.	P2
**METHODS**			
Eligibility criteria	5	Specify the inclusion and exclusion criteria for the review and how studies were grouped for the syntheses.	P3
Information sources	6	Specify all databases, registers, websites, organisations, reference lists and other sources searched or consulted to identify studies. Specify the date when each source was last searched or consulted.	P3
Search strategy	7	Present the full search strategies for all databases, registers and websites, including any filters and limits used.	Appendix B
Selection process	8	Specify the methods used to decide whether a study met the inclusion criteria of the review, including how many reviewers screened each record and each report retrieved, whether they worked independently, and if applicable, details of automation tools used in the process.	P3-4
Data collection process	9	Specify the methods used to collect data from reports, including how many reviewers collected data from each report, whether they worked independently, any processes for obtaining or confirming data from study investigators, and if applicable, details of automation tools used in the process.	P3-4
Data items	10a	List and define all outcomes for which data were sought. Specify whether all results that were compatible with each outcome domain in each study were sought (e.g. for all measures, time points, analyses), and if not, the methods used to decide which results to collect.	P3-4
	10b	List and define all other variables for which data were sought (e.g. participant and intervention characteristics, funding sources). Describe any assumptions made about any missing or unclear information.	P3-4
Study risk of bias assessment	11	Specify the methods used to assess risk of bias in the included studies, including details of the tool(s) used, how many reviewers assessed each study and whether they worked independently, and if applicable, details of automation tools used in the process.	P3-4
Effect measures	12	Specify for each outcome the effect measure(s) (e.g. risk ratio, mean difference) used in the synthesis or presentation of results.	P3-4
Synthesis methods	13a	Describe the processes used to decide which studies were eligible for each synthesis (e.g. tabulating the study intervention characteristics and comparing against the planned groups for each synthesis (item #5)).	N/A
	13b	Describe any methods required to prepare the data for presentation or synthesis, such as handling of missing summary statistics, or data conversions.	N/A
	13c	Describe any methods used to tabulate or visually display results of individual studies and syntheses.	N/A
	13d	Describe any methods used to synthesize results and provide a rationale for the choice(s). If meta-analysis was performed, describe the model(s), method(s) to identify the presence and extent of statistical heterogeneity, and software package(s) used.	P3-4
	13e	Describe any methods used to explore possible causes of heterogeneity among study results (e.g. subgroup analysis, meta-regression).	N/A
	13f	Describe any sensitivity analyses conducted to assess robustness of the synthesized results.	N/A
Reporting bias assessment	14	Describe any methods used to assess risk of bias due to missing results in a synthesis (arising from reporting biases).	N/A
Certainty assessment	15	Describe any methods used to assess certainty (or confidence) in the body of evidence for an outcome.	N/A
**RESULTS**			
Study selection	16a	Describe the results of the search and selection process, from the number of records identified in the search to the number of studies included in the review, ideally using a flow diagram.	Fig. 1
	16b	Cite studies that might appear to meet the inclusion criteria, but which were excluded, and explain why they were excluded.	Fig. 1
Study characteristics	17	Cite each included study and present its characteristics.	P4-5
Risk of bias in studies	18	Present assessments of risk of bias for each included study.	P4-5
Results of individual studies	19	For all outcomes, present, for each study: (a) summary statistics for each group (where appropriate) and (b) an effect estimate and its precision (e.g. confidence/credible interval), ideally using structured tables or plots.	P4-5
Results of syntheses	20a	For each synthesis, briefly summarise the characteristics and risk of bias among contributing studies.	N/A
	20b	Present results of all statistical syntheses conducted. If meta-analysis was done, present for each the summary estimate and its precision (e.g. confidence/credible interval) and measures of statistical heterogeneity. If comparing groups, describe the direction of the effect.	N/A
	20c	Present results of all investigations of possible causes of heterogeneity among study results.	N/A
	20d	Present results of all sensitivity analyses conducted to assess the robustness of the synthesized results.	N/A
Reporting biases	21	Present assessments of risk of bias due to missing results (arising from reporting biases) for each synthesis assessed.	N/A
Certainty of evidence	22	Present assessments of certainty (or confidence) in the body of evidence for each outcome assessed.	N/A
**DISCUSSION**			
Discussion	23a	Provide a general interpretation of the results in the context of other evidence.	P5-6
	23b	Discuss any limitations of the evidence included in the review.	P5-6
	23c	Discuss any limitations of the review processes used.	P5-6
	23d	Discuss implications of the results for practice, policy, and future research.	P5-6
**OTHER INFORMATION**			
Registration and protocol	24a	Provide registration information for the review, including register name and registration number, or state that the review was not registered.	P3
	24b	Indicate where the review protocol can be accessed, or state that a protocol was not prepared.	Abstract and P3
	24c	Describe and explain any amendments to information provided at registration or in the protocol.	N/A
Support	25	Describe sources of financial or non-financial support for the review, and the role of the funders or sponsors in the review.	P6
Competing interests	26	Declare any competing interests of review authors.	P6
Availability of data, code and other materials	27	Report which of the following are publicly available and where they can be found: template data collection forms; data extracted from included studies; data used for all analyses; analytic code; any other materials used in the review.	P6

*From:* Page MJ, McKenzie JE, Bossuyt PM, Boutron I, Hoffmann TC, Mulrow CD et al. The PRISMA 2020 statement: an updated guideline for reporting systematic reviews. BMJ 2021; 372:n71. doi: 10.1136/bmj.n71.

## Appendices

Appendix PRISMA Checklist (Appendix A); Systematic reviews used to derive search terms; Search Terms and Syntax used (Appendix B).

### Appendix B Recent systematic reviews and bibliometric analyses of physical literacy studies used to derive the search terms; Search Terms and Syntax used

#### Systematic reviews and bibliometric analyses

Fang X, Zhang Z. Hotspots and trends in health-oriented physical literacy research: a visual analysis based on the WOS database. *BMC Public Health* 2024; *24*: 1480. https://doi.org/10.1186/s12889-024-18951-7.

Grauduszus M., Koch L, Wessely S, Joisten C School-based promotion of physical literacy in physical education: a scoping review. Front Publ Health 2024; 2: 13220.

Jerebine A, Arundell L, Watson-Mackie K et al. Effects of holistically conceptualized school-based interventions on children's physical literacy, physical activity and other outcomes: a systematic review. Sports Med Open 2024; 10:105.

Qian H, Ren X, Wang H, Zou Y. Global insights and key trends in physical literacy research: a bibliometric analysis and literature review. J Multidiscip Healthcare 2025; 18: 2039–2055.

Carl J, Barratt J, Töpfer C, Cairney J, Pfeifer K. How are physical literacy interventions conceptualized? – A systematic review on intervention design and content: Design/Content of Physical Literacy Interventions. In Psychology of Sport and Exercise. 2022; 58.https://doi.org/10.1016/j.psychsport.2021.102091.

#### Search Terms and Syntax in Web of Science

Physical∗literate OR physical literac∗

AND

Covid∗ OR isolat∗ OR lockdown∗ OR restrict∗ or pandemic∗ or coronavirus∗

AND

Child∗ or young people OR adolescen∗ OR youth∗ or pre-adolescen∗ OR teen∗ or young person∗
